# Immunohistochemical detection of transgene expression in the brain using small epitope tags

**DOI:** 10.1186/1472-6750-10-16

**Published:** 2010-02-18

**Authors:** Evy Lobbestael, Veerle Reumers, Abdelilah Ibrahimi, Kirsten Paesen, Irina Thiry, Rik Gijsbers, Chris Van den Haute, Zeger Debyser, Veerle Baekelandt, Jean-Marc Taymans

**Affiliations:** 1 Laboratory for Neurobiology and Gene Therapy, Division of Molecular Medicine, Department of Molecular and Cellular Medicine, Katholieke Universiteit Leuven, Leuven, Belgium; 2 Laboratory for Molecular Virology and Gene Therapy, Division of Molecular Medicine, Department of Molecular and Cellular Medicine, Katholieke Universiteit Leuven, Leuven, Belgium; 3 Molecular small animal imaging center (MoSAIC), Katholieke Universiteit Leuven, Leuven, Belgium

## Abstract

**Background:**

*In vivo *overexpression of proteins is a powerful approach to study their biological function, generate disease models or evaluate gene therapy approaches. In order to investigate an exogenously expressed protein, specific and sensitive detection is essential. Unfortunately, antibodies that allow histological detection of the protein of interest are not always readily available. The use of an epitope tag fused to the protein can circumvent this problem as well as provide the possibility to discriminate endogenous from overexpressed proteins. In order to minimize impact on the bioactivity and biodistribution of the overexpressed protein, preference is given to small tags.

**Results:**

In the present study, we evaluated several small epitope tags together with corresponding anti-tag antibodies for the detection of overexpressed proteins in rat brain, using eGFP as a reference. We generated several lentiviral vectors encoding eGFP with different N-terminally fused small epitope tags (AU1, flag, 3flag, HA, myc and V5). After confirmation of their functionality in cell culture, we injected these lentiviral vectors stereotactically into the striatum of rats and prepared paraformaldehyde fixed floating sections for immunohistochemical analysis. Using multiple antibodies and antibody dilutions per epitope tag, we extensively assessed the efficiency of several anti-tag antibodies for chromogenic immunohistochemical detection of the epitope tagged eGFPs by determining the proportion of immunoreactivity detected by anti-tag antibodies compared to anti-GFP antibody. Using fluorescence immunohistochemistry and confocal microscopy, we also quantified the proportion of eGFP-positive cells detected by anti-tag antibodies. Our results show that all the examined small epitope tags could be detected by anti-tag antibodies both in cell extracts as well as *in vivo*, although to varying degrees depending on the tag and antibody used. Using the presented protocol, V5/anti-V5 and HA/HA11 tag/antibody combinations provided the most sensitive detection in brain tissue. We confirmed the applicability of these optimized *in vivo *tag detection conditions for a difficult to detect protein, firefly luciferase (fLuc), using lentiviral vector constructs expressing V5 tagged and 3flag tagged fLuc protein.

**Conclusions:**

We show here that several small epitope tags are useful for immunohistochemical detection of exogenous proteins *in vivo*. Our study also provides a generic methodology which is broadly applicable for the detection of overexpressed transgenes in mammalian brain tissue.

## Background

Since the advent of recombinant DNA technology, transgenic model organisms have become powerful tools for the study of the basic biology of proteins or to generate *in vivo *models for diseases [1]. The expression of transgenes in complex organisms is accompanied by the need for a specific and sensitive detection of the protein. One approach is the use of a protein specific antibody. However, antibodies raised against a protein of interest are not always available, are costly and time-consuming to produce and are usually not transgene specific. Moreover antibodies are often not suitable for several applications and immunohistochemical detection is a frequent bottleneck. These drawbacks can be overcome by the use of epitope tagging. The fusion of an immunoreactive epitope tag to a protein provides the possibility to detect any transgene product in a very specific and sensitive manner with well-characterized commercially available antibodies. Moreover, it allows discriminating endogenous from overexpressed proteins.

The performance of an epitope tag in a detection experiment is dependent not only on the epitope used but also on the anti-epitope antibody [2]. The selection of the optimal tag/antibody combination is complicated and depends on the target protein and the application. The large variety of combinations allows selecting the appropriate tag/anti-tag antibody for a particular application; however this optimization may be a time-consuming process. Despite the extensive documentation on the use of epitope tagging for *in vitro *or cellular applications, very little information is available concerning the use of epitope tags for *in vivo *applications [2].

In the comparative study presented here, we aimed to characterize different commonly used small epitope tag/antibody combinations in cell culture as well as *in vivo*. In the selection of different tags, preference was given to those tags with broad versatility: AU1 [3], HA [4,5], myc [6], V5 [7], flag and 3flag [8]. In order to evaluate the different tag/antibody combinations, epitope tags were N-terminally fused to eGFP and overexpressed by means of locoregional lentiviral vector-mediated gene transfer [9]. We evaluated detection of the epitope tags in cell extracts as well as in the rat striatum in comparison to detection of eGFP. As proof-of-principle, we evaluated the indirect detection of fLuc protein fused to a V5 or 3flag tag after locoregional overexpression in the mouse striatum.

## Results

### Evaluation of epitope tag expression in cell extracts

**Figure 1:**
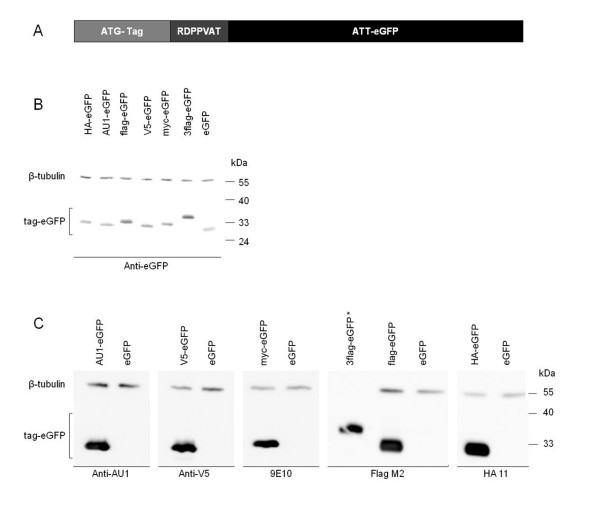
**SDS-PAGE analysis of tag-eGFP fusion proteins in LV transduced HEK293T cells**. A) Schematic representation of the tag-eGFP constructs. Peptide sequences are listed in table 1. B) Overexpressed proteins were detected with an in-house anti-eGFP antibody. C) The same lysates as in B) were analyzed with the different anti-tag antibodies. 2 μg of cell lysate was loaded and antibody dilutions were as follows: anti-eGFP (1/10000), anti-AU-1 (1/25000), anti-V5 (1/250000), 9E10 (1/10000), FlagM2 (1/30000) and HA 11 (1/50000). β-tubulin was used as loading control. * cell lysate of 3flag-eGFP was loaded at 4 ng to avoid saturation of the western blot signal.

HEK293T cells were transduced with lentiviral vectors encoding different tag-eGFP fusion proteins or eGFP alone (Table 1 and Fig. 1A). The amount of vector was normalized for expression based on functional titers (transducing units; TUs) as described in materials and methods. Western blot analysis confirmed a clear expression and detection of all different fusion proteins and eGFP to comparable levels. Our in-house anti-eGFP antibody [9] as well as the different commercially available antibodies detected the appropriate fusion protein with high specificity. As shown in Fig. 1, no notable proteolytic cleavage of the fusionproteins was observed. In order to avoid saturation of the western blot signal, the anti-V5 antibody had to be used at a 1:250000 dilution, indicating a very sensitive detection of V5-eGFP by this antibody. To obtain bands with comparable intensity for cell lysates overexpressing 3flag-eGFP and flag-eGFP; the former had to be diluted 500 times, showing increased detection efficiency of the 3flag tag compared to flag alone.

**Table 1:** Overview of evaluated small epitope tags

Tag	Sequence
Triple flag	DYKDHDGDYKDHDIDYKDDDDK
flag	DYKDDDDK
V5	GKPIPNPLLGLDST
myc	EQKLISEEDL
HA	YPYDVPDYA
AU1	DTYRYI

Peptide sequences of evaluated small epitope tags. All tags were N-terminally fused to an eGFP sequence in which the start codon had been replaced, a RDPPVAT sequence was used as linker. Untagged eGFP was included as control.

### Evaluation of epitope tag/antibody combinations *in vivo*

Using the same lentiviral vectors, stereotactic injections in striatum of adult rats were performed. The use of tag-eGFP fusion proteins allowed us to use eGFP detection levels as internal control. Animals were sacrificed 2 weeks post-injection, a time point at which expression levels reach a maximum [10]. Adjacent sections were subjected to DAB-based immunohistochemical staining with our in-house anti-eGFP antibody or the different tag-specific antibodies. Dilutions were optimized as described in materials and methods to obtain the optimal signal-to-noise ratio (Table 2).

**Table 2:** Detailed overview of characterized antibodies

Epitope	Antibody	Clonality	Company	Product N°	Concentration	Optimal dilution for IHC based on current study	Optimal concentration (μg/ml) for IHC based on current study
**3flag and flag**	Anti-flag polyclonal^1^	Polyclonal	Sigma	F7425	0.8 mg/ml	1/5000	0.16
	FlagM2^1^	Monoclonal	Sigma	F3165	5 mg/ml	1/12500	0.4
**V5**	Anti-V5^3^	Monoclonal	Invitrogen	R960-25	1.07 mg/ml	1/12500	0.0856
**Myc**	Anti-Myc^2^ Polyclonal	Polyclonal	Upstate	06-549	1 mg/ml	1/5000	0.2
	9E10^3^	Monoclonal	Santa Cruz	Sc-40	0.2 mg/ml	1/1000	0.2
**HA**	HA 11^3^	Monoclonal	Covance	MMS-101R	2-3 mg/ml	1/25000	0.1
	12CA5^3^	Monoclonal	Roch Applied	11 583 816 001	0.4 mg/ml	1/5000	0.08
**AU1**	Anti-AU1^3^	Monoclonal	Covance	MMS-130R	5-7 mg/ml	1/5000	1.2
**GFP**	Anti-eGFP^4^ Polyclonal	Polyclonal	In-house	-	-	1/10000	-

^1 ^affinity purified IgGs using a column bearing the inmmunizing peptide

^2 ^affinity purified IgGs using a column bearing protein A

^3 ^purification method not specified

^4 ^not purified

In order to compare antibody dilutions, the optimal dilutions (based on current study) for IHC reckoned with the antibody concentration are mentioned in this table.

**Figure 2:**
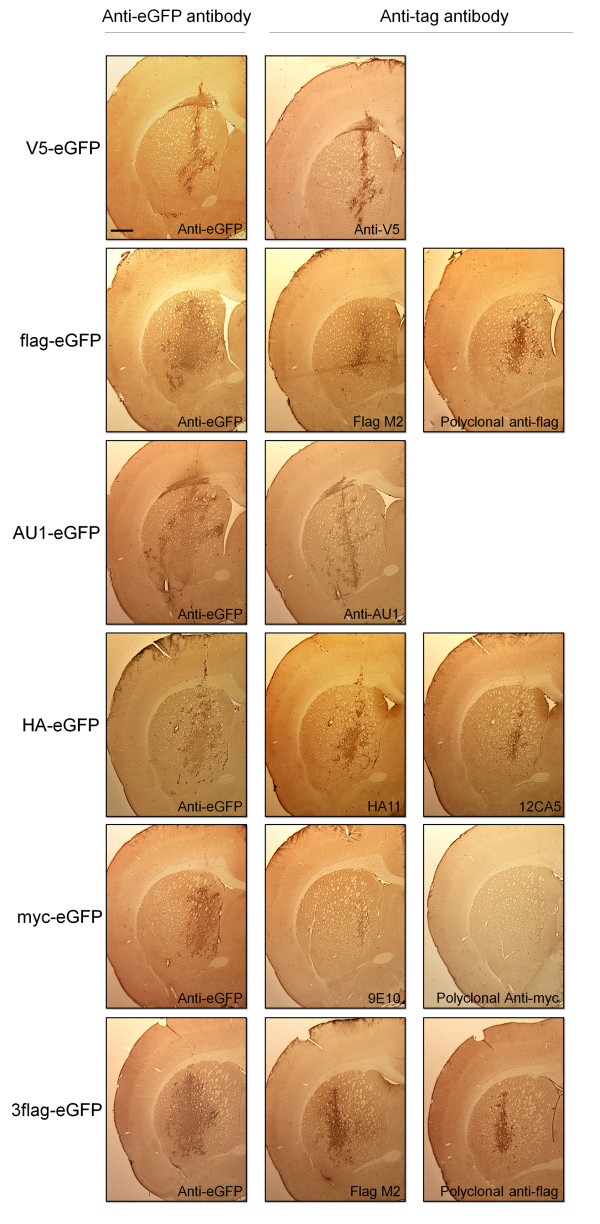
***In vivo *detection of tag-eGFP constructs**. Rats were stereotactically injected in the striatum with lentiviral vectors encoding the tag-eGFP fusion protein. The overexpressed protein (indicated on the left) was detected in adjacent sections with either the anti-eGFP antibody or the anti-tag antibody as indicated. Antibody dilutions used are given in table 2. Scale bar: 1 mm.

**Figure 3:**
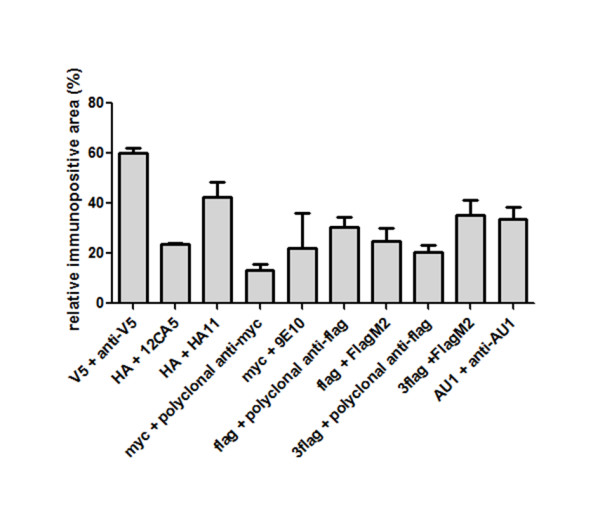
**Quantification of detection of tag-eGFP constructs**. Proportion of the transduced surface detected by the anti-tag antibody compared to the surface detected by the anti-eGFP antibody on adjacent slides. Three slides through the transduced region were quantified per animal with n = 3 per condition. Results are expressed as mean percentages +/- the standard error of the mean.

Comparison of the transduced regions revealed that all antibodies can detect the corresponding tag-eGFP fusion protein to varying degrees, although the maximum detection levels reached by the evaluated anti-tag antibodies were in all conditions lower than the corresponding eGFP detection with our in-house anti-eGFP antibody (Fig. 2). In order to evaluate the sensitivity of the different tag/antibody combinations, we determined the ratio of tag immunopositive surface to eGFP immunopositive surface through the transduced region (Fig. 3). This quantification demonstrates that the V5/anti-V5 and HA/HA11 combinations show the highest sensitivity. The polyclonal anti-myc antibody on the other hand detects its antigen, under the conditions tested, with very low sensitivity.

**Figure 4:**
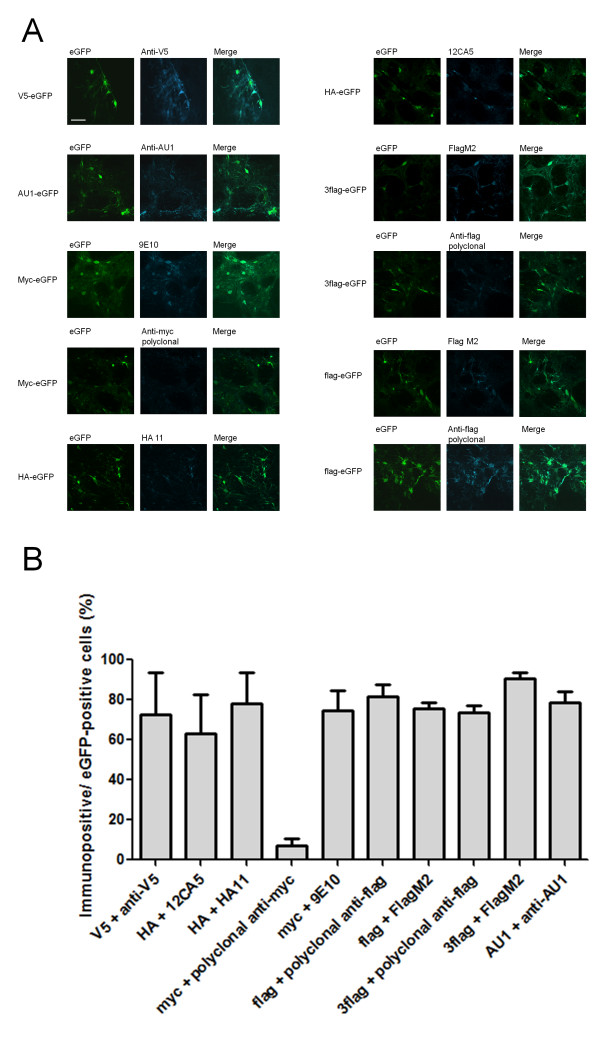
**Detailed immunofluorescent detection of overexpressed tag-eGFP *in vivo***. Adjacent sections of the analyzed tissue described in Fig. 2 and 3 were immunofluorescently stained with anti-tag antibody using the dilutions given in table 2. A) A representative picture is shown for each tag/antibody combination, the left part shows eGFP fluorescence, the middle part shows the tag staining and the right part is a merged picture of the two individual pictures. Overlay pictures reveal the degree of anti-tag antibody sensitivity. Scale bar: 50 μm. B) Mean percentage transduced cells recognized by the specific anti-tag antibody for each tag. Twelve images per animal (n = 3) were counted per epitope tag. Results are expressed as means with standard error of the mean.

Next, fluorescent immunohistochemical stainings were performed to further characterize detection sensitivity as well as to evaluate specificity at the cellular level (Fig. 4A). Panels with eGFP show primary eGFP fluorescence without additional staining, detection with the tag-specific antibodies was performed as described in materials and methods. This data confirms that all antibodies can detect the corresponding tag *in vivo*. Quantification of the percentage of eGFP-positive cells that stained positive using the corresponding anti-tag antibody were performed within the centre of the transduced region (Fig. 4B). In this area, where transgene expression levels are high, all but one of the evaluated tag specific antibodies detected the corresponding protein with a similar sensitivity between 70 - 90%. The polyclonal anti-myc antibody could only detect 10% of the eGFP-positive cells. Aspecific detection, i.e. presence of tag immunoreactivity that was not eGFP positive, was negligible in all cases.

### Detection of firefly luciferase using epitope tagging

**Figure 5:**
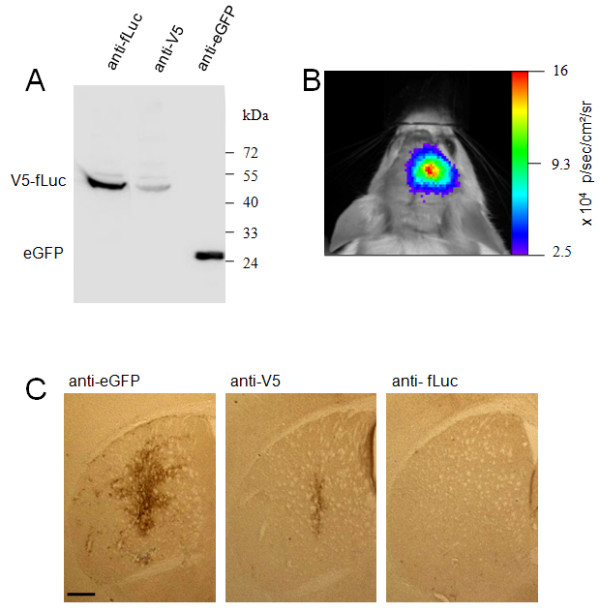
**Detection of V5-fLuc after intrastriatal LV injection in mice**. As proof of principle experiment, V5 was fused to fLuc in order to facilitate the transgene detection. A) HEK293T cell lysates transduced with LV encoding V5-fLuc-T2A-eGFP were analyzed by SDS-PAGE. The overexpressed fusion protein was detected with both the anti-fLuc antibody (1/3000) and the anti-V5 antibody (1/250000). The co-expressed eGFP was detected by our in-house anti-eGFP antibody (1/10000). B) The same LV as in A) was stereotactically injected in mice striatum. Luciferase activity was confirmed by non-invasive BLI four days and two weeks after injection. A representative image of luciferase activity 4 days post-injection is shown. C) Striatal sections of the same mice as described in B) were immunohistochemically stained with anti-eGFP (1/10000), anti-V5 (1/500) and anti-fLuc antibody (1/500). Scale bar: 0.5 mm.

As proof of principle, we tested immunohistochemical detection of tagged firefly luciferase (fLuc) following locoregional overexpression in mouse striatum. Luciferase, frequently used as *in vivo *imaging reporter gene, can generate photons in the presence of its substrate D-luciferin and ATP. These photons can be detected by a light sensitive device. However, immunohistochemical confirmation of luciferase expression is often crucial. Although anti-luciferase antibodies are commercially available, their sensitivity for immunohistochemical detection in brain is very low [11]. As the V5 tag/anti-V5 antibody showed the highest sensitivity for V5-eGFP *in vivo*, we tested the detection of V5-tagged luciferase. As a control for transduced cells, V5-luciferase was co-expressed with eGFP using a T2A peptide sequence [12]. The western blot presented in Fig. 5A confirms expression of our construct. The V5-luciferase fusion protein could be detected with both the luciferase antibody and the anti-V5 antibody in cellular extracts. To evaluate the detection of V5-luciferase *in vivo*, we injected the lentiviral vector encoding V5-luciferase-T2A-eGFP in the striatum of mice. To assess the expression, functionality and stability of luciferase protein after lentiviral transduction, mice were imaged by bioluminescence imaging (BLI) at 4 days and 2 weeks post-injection (1.28E+06 +/- 8.30E+05 and 6.29E+05 +/- 2.65E05) (Fig. 5B). Images of both time points demonstrated a signal originating from the injected side of the brain. Animals were sacrificed 2 weeks post-injection and brains were processed for immunohistochemical analysis. In order to evaluate detection efficiency, adjacent sections were incubated with either the anti-V5 antibody, anti-luciferase antibody or the in-house anti-eGFP antibody (Fig. 5C). Despite confirmation of luciferase activity by BLI, the anti-luciferase antibody failed to detect the transgene, while the anti-V5 antibody clearly detected V5-luciferase in the transduced region visualized by eGFP.

## Discussion

Exogenous gene transfer into the brain is an important strategy to study *in vivo *protein function, investigate pathogenic mechanisms of diseases as well as develop new therapeutic strategies. However, the study of exogenously expressed proteins requires rigorous assessment of transgene expression, preferentially distinct from the corresponding endogenous protein. In this study, we have used lentiviral vector-based expression of small epitope tags (AU1, flag, 3flag, myc, V5, HA) N-terminally fused to eGFP in rat brain to evaluate immunohistochemical detection of several commonly used epitope tags in floating sections. In this way, we have evaluated the performance of several commonly used epitope tag/antibody combinations.

First, we evaluated the detection of the different epitope tags in HEK293T cell extracts after transduction with lentiviral vectors encoding the different tag-eGFP fusion proteins. Western blot analysis shows a distinct and specific detection using our in-house anti-eGFP antibody as well as all examined anti-tag antibodies. Especially the V5/anti-V5 antibody and 3flag/FlagM2 antibody combinations allow a very sensitive detection in cell extracts (Fig. 1C).

The main goal of this study was to compare *in vivo *detection of different frequently used epitope tags using commercially available antibodies. Chromogenic DAB-based as well as fluorescent immunohistochemical detection of the overexpressed tags revealed that all epitope tag/antibody combinations, with the exception of myc/polyclonal anti-myc, meet the requirements of a specific and sensitive detection in rat brain (Fig. 2, 3 and 4). The specificity of the V5/anti-V5 and HA/HA11 combinations are comparable with the other combinations; however the sensitivity seems to be higher since lower antibody concentrations were used for optimal immunohistochemical detection (Fig. 3 and Table 2). These differences were most apparent for the immunohistochemical detection with DAB. The polyclonal anti-myc antibody on the other hand, detects its antigen with low sensitivity as well as low specificity. Therefore, using the presented protocol, our findings show that all tag/antibody combinations, with the exception of myc/polyclonal anti-myc, show sufficient sensitivity and specificity for immunohistochemical analysis in brain tissue.

Based on the strong increase in sensitivity in western blotting detection, we expected the detection of the 3flag tag *in vivo *to be more sensitive compared to the flag tag; however no significant differences in sensitivity were observed between these two tags using either the monoclonal FlagM2 antibody or a polyclonal anti-flag antibody.

In terms of sensitivity, our in-house eGFP antibody was superior to all tested tag antibodies. However by using a large protein such as eGFP itself as tag for a protein of interest, caution should be exercised since eGFP fusion might interfere with the normal protein function, especially for small proteins. An alternative strategy could consist of a bicistronic construct using a T2A-like peptide [12], although a minor fraction of fusion protein might be produced with this system.

As proof-of-principle experiment, we overexpressed firefly luciferase tagged with V5 and co-expressed with eGFP in mouse striatum. Luciferase activity was confirmed via bioluminescence imaging. The enzymatic activity of fLuc was not affected by tagging with V5 since the BLI signal per injected transducing unit vector does not differ from untagged fLuc (3,3 p/s/TU for V5-fLuc versus 2,3 p/s/TU for untagged fLuc). However, immunohistochemical analysis using a commercial anti-luciferase antibody failed to detect the transgene. In contrast, we were able to clearly detect the V5-luciferase using the V5 antibody (Fig. 5). The detection sensitivity with the V5 antibody appears somewhat lower than in the case of the V5-eGFP fusion protein (Fig. 2). This may be attributed to differences in stability of the two proteins (eGFP_T1/2_: >10 h, fLuc_T1/2_: 3 h) since initial protein levels of V5-luciferase and eGFP are identical when co-expressed using a T2A sequence [12]. This example clearly underlines the advantage of epitope tagging for sensitive and specific detection of an exogenous protein which is otherwise not readily detectable with IHC.

In order to confirm the previous data with another tag, 3flag-luciferase-T2A-eGFP was injected and BLI signals were measured. We could confirm that the activity of tagged fLuc is maintained and even slightly increased (157,20 p/s/TU for 3flag-fLuc versus 38,84 p/s/TU for untagged fLuc). We therefore conclude that tagging of luciferase with either a V5 or 3flag tag does not adversely affect sensitivity of bioluminescence imaging in the brain.

One of the main concerns fusing a small epitope tag to a protein of interest is the potential impact on properties such as biodistribution, stability, enzymatic activity and binding capacity. Since such effects cannot be predicted, control studies comparing the tagged protein with the wild-type counterpart should always be included [13-15]. However, interference with the tertiary structure and bioactivity can be minimized by using short peptide sequences fused to the terminus of the protein [16].

Besides immunohistochemical detection evaluated in the present study, fusing a small peptide to any gene of interest also offers great benefit in several other applications. For instance, epitope tags can be used as an affinity tag to perform protein purification when using stringent purification conditions or to identify interacting proteins when combining non-stringent affinity purifications and mass spectrometry analysis of co-purified proteins [16-27]. Moreover, modifications of the small peptide sequence may further enlarge the range of applications [28]. An interesting new development is for example the generation of a drug-controllable tag [29].

It should be noted that since the choice of tag/antibody combinations is endless and the ideal epitope tag may vary for every transgene and every application, there is still a need for optimization and characterization studies when tagging a new protein [17,30]. The evaluation of several commonly used tag/antibody combinations *in vivo *presented here can be used as an aid in the selection of tags to assess in priority. Moreover, we offer a straightforward protocol for immunohistochemical analysis of tagged exogenous protein expressed in rodent brain which can be readily applied for any new protein of interest.

## Conclusions

In conclusion, the present study provides an extensive overview of small epitope tags and corresponding antibodies for immunohistochemical detection of transgenes *in vivo*. Using the presented protocol, V5/anti-V5 and HA/HA11 are the preferable tag/antibody combinations for immunohistochemical analysis in brain tissue while detection of myc-tagged proteins by polyclonal anti-myc antibody is not an ideal candidate. Our study yields a straightforward protocol for sample preparation and immunohistochemical staining in floating sections of brain tissue. We show in this study that tagging a protein with a small epitope tag is a powerful approach to obtain sensitive and specific detection of overexpressed proteins while reducing the risks of altering the transgene's function.

## Methods

### Cloning

The different epitope tags AU-1 (6 amino acids (aa)), flag (8 aa), 3flag (22 aa), HA (9 aa), myc (10 aa) and V5 (14 aa) were N-terminally fused to eGFP (Tables 1 and 2 and Fig. 1A) by adaptor ligation in a peGFP-N1 plasmid (Clontech, Mountain View, CA, USA). All tagged eGFP constructs contained a linker sequence between tag and eGFP of RDPPVAT. To avoid eGFP expression alone, we mutated the startcodon of eGFP to ATT via a PCR based method [31]. These fusion DNA sequences, as well as eGFP alone, were subcloned into the pCHMWS plasmid backbone, an in-house lentiviral transfer plasmid (as described in [10]) in which transgene expression is under control of a CMV promoter. V5-fLuc and 3flag-fLuc, followed by a T2A eGFP sequence, were also cloned into the pCHMWS backbone. The T2A sequence allows bicistronic gene expression since the presence of this viral peptide results in equimolar expression of V5-fLuc or 3flag-fLuc and eGFP [12]. All constructs were sequence confirmed.

### Lentiviral vector production

HIV-1-derived vector particles encoding the fusion proteins tag-eGFP, V5-luciferase-T2A-eGFP, 3flag-luciferase-T2A-eGFP or eGFP alone were produced, essentially as described in Ibrahimi et al. [12]. Briefly, after seeding HEK293T cells in a 10-cm dish, we performed a triple transient transfection with the respective transfer plasmids, a packaging plasmid and an envelope plasmid encoding VSV G. The production was performed in Opti-MEM I (Gibco-Invitrogen, Merelbeke, Belgium). The medium was replaced after 24 h. Cell supernatant containing lentiviral vectors was collected on day 2 and 3 post-transfection, filtered through a 0.45 μm pore size filter (Sartorius, Minisart, Göttingen, Germany) and concentrated 50-fold using a Vivaspin 15 column (Vivascience, Hannover, Germany).

Functional vector titers were determined by transducing HEK293T cells with the respective vector preparations in a 10-fold dilution series. Three days after transduction, cells were harvested and fixed in 4% paraformaldehyde (PAF) and analysed using a FACSCalibur flow cytometer (BD Biosciences, Erembodegem, Flanders) and the CellQuest software package provided with the instrument. These functional vector titers are expressed as transducing units (TUs) per ml.

### Antibodies

Table 2 shows an overview of the antibodies used in the present study. These antibodies were used for detection via western blotting as well as IHC. To determine the optimal antibody dilution for immunohistochemical evaluation, three different dilutions were tested: the antibody manufacturer's recommended dilution as well as a 5-fold lower and 5-fold higher dilution. Depending on the origin of the primary antibody, goat-anti-mouse or goat-anti-rabbit secondary antibodies were used.

### Cell culture and transduction

HEK293T cells were maintained in Dulbecco's modified Eagle's medium (Gibco) supplemented with 10% heat-inactivated fetal calf serum (Harlan Sera-Lab Ltd., International Medical, Brussels, Belgium) and 50 μg/ml gentamycin at 37°C in a humidified atmosphere containing 5% CO_2_.

Cells were seeded in a 24 well plate at 200000 cells/well. 24 h after seeding, cells were transduced using 10^6 ^TUs/ml vector. After 48 h, cells were harvested and lysed in 1% sodium dodecyl sulphate (SDS) lysis buffer containing protease inhibitor cocktail (Sigma-Aldrich, Bornem, Belgium).

### Western blot analysis

Protein content of cell lysates was determined using the bicinchoninic acid (BCA) protein determination assay (Pierce Biotechnology, **Rockford, IL, USA**). Cell lysates were boiled in 1% SDS sample buffer containing protease inhibitor cocktail for 5 minutes (min). 2 μg of each total protein extract was resolved on a 12.5% polyacrylamide gel. Separated proteins were transferred to a polyvinylidene fluoride membrane and aspecific binding sites were blocked for 30 min in PBS supplemented with 0.1% Triton X-100 (PBST) and 5% non-fat milk. After overnight incubation at 4°C with the appropriate antibody, blots were washed 3 times with PBST. An anti-mouse beta-tubulin antibody was used as loading control. After incubation with the appropriate horseradish peroxidase-labeled secondary antibody, blots were again washed as mentioned before. Bands were visualized using enhanced chemiluminescence (Amersham Pharmacia Biotech, Little Chalfont, England).

### Stereotactic injections and perfusion

Housing and handling of rats and mice were done in compliance with national guidelines; all animal procedures used were approved by the Institutional Care and Use Committee of the Katholieke Universiteit Leuven. All experiments involving viral vectors were carried out under biosafety level 2 conditions.

Eight week old female Wistar rats were used for tag-eGFP injections, while eight week old female C57BL/6-*Tyrc-2J*/J mice were used for BLI. The animals were anaesthetized and placed in a stereotactic head frame. After making a midline incision of the scalp, a burr hole was drilled in the appropriate location at both sites of the skull using bregma as reference. Following coordinates were used for mouse and rat respectively: anteroposterior 0.5 and 0 mm; lateral 2.0 and 2.8 mm; dorsoventral 3.0 and 5.5 mm. Three microliters (corresponding to 1.26 E+04 to 5.67 E+04 TU) of lentiviral vector supplemented with polybrene (to 4 μg/ml) was injected in rat striatum at a rate of 0.25 ml/min with a 30-gauge needle on a 10-ml Hamilton syringe. Mice were injected with 2 microliters lentiviral vector. After the injection, the needle was left in place for an additional 5 min before being slowly withdrawn from the brain (adapted from [32]). Two weeks later, animals were deeply anesthetized using an overdose of pentobarbital and transcardially perfused with saline followed by ice-cold 4% PAF in PBS. The brain was removed from the skull and post-fixed overnight in 4% PAF-PBS at 4°C.

### Histology

50-mm-thick coronal brain sections were cut with a microtome (HM650V, Microm, Walldorf, Germany) and stored at 4°C in PBS with 0.1% sodium azide. The transduced area was identified based on eGFP expression using an inverted fluorescence microscope (Leica DMR optical microscope, Microsystems, Wetzlar, Germany). Sections around this injection area were used for histology with the different tag/antibody combinations.

Polyclonal in-house rabbit anti-eGFP antibody [9], a small epitope anti-tag antibody (Table 2) or anti-luciferase antibody (Promega, Madison, WI, USA) was used to detect the transgene. Chromogenic immunostaining was performed using an avidine-biotine-peroxidase complex immunostaining technique and carried out under uniform conditions. To remove endogenous peroxidase activity, sections were incubated with 3% hydrogen peroxide in PBS for 10 min. Non-specific sites were blocked by pre-treatment with 10% normal goat serum in PBST. Sections were then incubated overnight at room temperature with the appropriate primary antibody diluted in PBST with 10% goat serum as described in table 2. After being washed 3 times 5 min with PBST, the sections were incubated for 30 min at room temperature with goat anti-rabbit or anti-mouse biotinylated secondary antibody (Dako, Glostrup, Denmark) diluted at 1:300 in PBST. The sections were washed in PBST 3 times 5 min and incubated with Strept-ABC-HRP complex (Dako) for 30 min. After another wash step, detection was performed with diaminobenzidine (DAB) with H_2_O_2 _as substrate. The sections were coverslipped on gelatin-coated slides with DPX (Fluka, Bornem, Belgium).

For fluorescent staining, sections were incubated overnight with the appropriate primary antibodies diluted in PBST, 10% sodium azide and 10% goat serum. After three PBST rinses, sections were incubated for 2 hours at room temperature with goat anti-rabbit or goat anti-mouse IgG-Alexa 633 (diluted 1: 500, Invitrogen Molecular Probes). Sections were again rinsed with PBST and mounted on microscope slides with polyvinyl alcohol (Mowiol; Merck, La Jolla, CA, USA).

Sensitivity and specificity of the staining were quantified as described below under 'microscopy analysis'.

### *In vivo *bioluminescence imaging

BLI of fLuc activity was performed as described in Deroose et al. [11]. Shortly, mice were imaged in an IVIS 100 system (Xenogen, Alameda, CA, USA). Anaesthesia was induced in an induction chamber with 2.5% isoflurane in 100% oxygen and maintained in the IVIS imaging chamber. The mice were injected intravenously with D-luciferin (126 mg/kg, Xenogen) dissolved in PBS (15 mg/ml). Subsequently, they were placed in the prone position in the IVIS and 1 min frames were acquired until the maximum signal was reached. The data are reported as the photon flux (p/s) from a 1.6 cm^2 ^circular region of interest around the head.

### Microscopy analysis

DAB staining of transgene expression in brain sections was visualized by a Leica DMR optical microscope. In order to quantify the sensitivity, we determined the ratio of transduced surface detected by the anti-tag antibody to the surface detected by the anti-eGFP antibody on the adjacent slide. Three slides through the transduced region were quantified per animal with n = 3 per condition.

Fluorescence staining was visualized by confocal microscopy using a LSM510 Laser Scanning Microscope (Zeiss, Zaventem, Belgium). To determine the proportion of transduced cells recognized by the corresponding antibody, we compared the number of cells recognized by the anti-tag antibody with the number of eGFP-positive cells. Counts were based on 12 randomly taken confocal images per animal (with n = 3) within the centre of the transduced region (as in Fig. 4A). Photos were cropped and adjusted for contrast and brightness with Adobe Photoshop version 6.0 (Adobe Systems Incorporated, San Jose, CA, USA).

## Authors' contributions

EL, KP, IT, J-MT performed experiments with tagged eGFP constructs. AI, VR, IT, EL performed experiments with the V5-fLuc-T2A-eGFP and 3flag-fLuc-T2A-eGFP constructs. RG, AI, CVDH, ZD, VB provided advice on the preparation of lentiviral vectors and experimental setup. J-MT designed the study. EL, J-MT, VR and VB wrote the manuscript. All authors have read and approved the final manuscript. The authors declare no conflict of interest.
